# Geographic variation and risk factors for systemic and limb ischemic events in patients with symptomatic peripheral artery disease: Insights from the REACH Registry

**DOI:** 10.1002/clc.22721

**Published:** 2017-05-18

**Authors:** Jérémie Abtan, Deepak L. Bhatt, Yedid Elbez, Emmanuel Sorbets, Kim Eagle, Christopher M. Reid, Iris Baumgartner, David Wu, Mary E. Hanson, Hakima Hannachi, Puneet K. Singhal, Philippe Gabriel Steg, Gregory Ducrocq, for the REACH Registry Investigators

**Affiliations:** ^1^ FACT (French Alliance for Cardiovascular Trials), French Clinical Research Infrastructure Network, University Hospital Departments‐FIRE, Hôpital Bichat, Public Assistance Hospitals of Paris, Paris Diderot University Sorbonne University Paris Cité, and National Institute of Health and Medical Research U‐1148 Paris France; ^2^ Heart and Vascular Center Brigham and Women's Hospital, and Harvard Medical School Boston Massachusetts; ^3^ Avicenne Hospital Hôpital Public Assistance Hospitals of Paris Paris France; ^4^ Cardiovascular Center University of Michigan Ann Arbor Michigan; ^5^ Monash University, Clayton, Victoria Australia and Curtin University Bentley Perth Western Australia; ^6^ University Hospital of Bern Bern Switzerland; ^7^ Merck & Co., Inc. Kenilworth New Jersey; ^8^ National Heart and Lung Institute, Imperial College School of Medicine, Royal Brompton Hospital Imperial College London United Kingdom

**Keywords:** Ischemic Risk, Peripheral Artery Disease, Vorapaxar

## Abstract

**Background:**

Patients with symptomatic peripheral artery disease (PAD) are at high risk of ischemic events. However, data about predictors of this risk are limited.

**Hypothesis:**

We analyzed baseline characteristics and 4‐year follow‐up of patients enrolled in the international REduction of Atherothrombosis for Continued Health (REACH) Registry with symptomatic PAD and no history of stroke/transient ischemic attack to describe annual rates of recurrent ischemic events globally and geographically.

**Methods:**

The primary outcome was systemic ischemic events (composite of cardiovascular death, myocardial infarction, or stroke) at 4 years. The secondary outcome was limb ischemic events (composite of lower limb amputation, peripheral bypass graft, and percutaneous intervention for PAD) at 2 years. Multivariate analysis identified risk factors associated with recurrent ischemic events.

**Results:**

The primary endpoint rate reached 4.7% during the first year and increased continuously (by 4%–5% each year) to 17.6% by year 4, driven mainly by cardiovascular mortality (11.1% at year 4). Japan experienced lower adjusted ischemic rates (P < 0.01) vs North America. Renal impairment (P < 0.01), congestive heart failure (P < 0.01), history of diabetes (P < 0.01), history of myocardial infarction (P = 0.01), vascular disease (single or poly, P < 0.01), and older age (P < 0.01) were associated with increased risk of systemic ischemic events, whereas statin use was associated with lower risk (P = 0.03). The limb ischemic event rate was 5.7% at 2 years.

**Conclusions:**

Four‐year systemic ischemic risk in patients with PAD and no history of stroke or transient ischemic attack remains high, and was mainly driven by cardiovascular mortality.

## 1 INTRODUCTION

Over 202 million people worldwide were estimated to be living with peripheral artery disease (PAD) in 2010, with estimated prevalence rates of 9% in North America and 11% in Europe.^1^ The spectrum of PAD includes acute and chronic limb ischemia, asymptomatic PAD, claudication, and critical limb ischemia.^2^, ^3^ PAD is a strong predictor of myocardial infarction (MI), stroke, and death from vascular causes,^4^, ^5^ with an annual incidence of approximately 5% for the composite endpoint of stroke, MI, and death in patients with PAD over 1 year.^6^ Understanding the current trends in PAD prevalence and risk factors is critical in guiding preventive strategies to reduce the burden of PAD. However, contemporary, real‐world data regarding patient profiles, treatment patterns, and cardiovascular (CV) risks for PAD patients beyond 1 year are insufficient, and are often limited to a single geographic region.

To address gaps in evidence for characterization of longer‐term ischemic risk in PAD patients, we analyzed 4‐year data from the REduction of Atherothrombosis for Continued Health (REACH) Registry, an international registry of atherothrombosis^7^, ^8^, ^9^ in patients with symptomatic PAD with no history of stroke or transient ischemic attack (TIA), focusing on both systemic (MI, stroke, and CV death) and limb ischemic complications (lower limb amputation, peripheral bypass graft, and percutaneous intervention for PAD). Patients with prior stoke or TIA were excluded, because the risk and benefit balance of antithrombotic agents in this population is specific and has been previously published as a separate analysis.^10^ The objectives of the present study were to (1) describe annual rates of systemic ischemic events (MI, stroke, and CV death) over 4 years globally and by geographic region, and to identify associated risk factors; and (2) to describe limb ischemic event rates (a composite of lower limb amputation, peripheral bypass graft, and percutaneous intervention for PAD) over 2 years.

## 2 METHODS

### 2.1 Population

The design, methods, and main results of the REACH Registry, an international, prospective, observational study, have been previously described.^7^, ^11^ Briefly, from December 2003 to June 2004, consecutive outpatients ages 45 years or older with established coronary artery disease (CAD), cerebrovascular disease, or PAD, or with at least 3 atherothrombotic risk factors were enrolled. Documented PAD was defined as 1 or more of the following: current intermittent claudication with ankle‐brachial index of less than 0.9 or a history of intermittent claudication together with a previous and related intervention such as angioplasty, stenting, atherectomy, peripheral arterial bypass graft, or other vascular intervention including amputation. Documented CAD was defined as 1 or more of the following: stable angina with documented CAD, history of unstable angina with documented CAD, history of percutaneous coronary intervention, history of coronary artery bypass graft surgery, or previous MI.

Data were collated centrally using standardized case report forms. The initial follow‐up period was 2 years, but centers were invited to participate in a 2‐year extension. Only patients with PAD and no history of stroke or TIA were included in the present analysis. Signed informed consent was obtained from all patients, and the institutional review board in each country approved the protocol.

The population was divided into 7 geographical regions: North America, including Canada and United States of America; Latin America, including Brazil, Chile, and Mexico; Western Europe, including patients from Austria, Belgium, Finland, France, Germany, Greece, the Netherlands, Portugal, Spain, Switzerland, and the United Kingdom; Eastern Europe, including patients from Hungary, Romania, Russia, and Ukraine; Middle East including patients from Israel and the United Arab Emirates; Asia including patients from China, Taiwan, Hong Kong, Malaysia, the Philippines, and Thailand; and Japan.

### 2.2 Outcomes

Following enrollment, detailed baseline characteristics, treatment, and outcomes were collected annually. Endpoints were not adjudicated and were based on physician report at the time of follow‐up. We analyzed 3 systemic ischemic outcomes over 4 years: nonfatal MI, nonfatal stroke, and CV death; and 3 limb ischemic outcomes at 2 years: lower limb amputation, peripheral bypass graft, and percutaneous intervention for PAD. Limb ischemic outcomes were not adjudicated, but were tracked on a declarative basis at the end of follow‐up. Consequently, due to missing data at 4 years, this endpoint was analyzed at 2 years.

The primary outcome was the composite of the 3 systemic ischemic events, and the secondary outcome was the composite of the 3 limb ischemic events. Other secondary outcomes of interest included CV death, MI, and stroke analyzed separately, as well as CV hospitalization.

Stroke was verified by a neurologist consultation or hospital records. CV death was defined as any MI or stroke followed by death in the next 28 days regardless of the cause, death from pulmonary embolism, heart failure, death following vascular surgery, death following a visceral or limb infarction, or any sudden death unless proven to be non‐CV by autopsy.

### 2.3 Statistical analysis

Descriptive statistics, including frequencies and percentages for categorical variables, and mean and standard deviation for continuous variables were calculated to describe the patients’ baseline characteristics, medical history, and treatment patterns using the overall study population. Kaplan‐Meier estimates were used to assess cumulative incidence rates at each year of follow‐up. Patients from each region were also investigated as subgroups.

The exact date of each systemic ischemic event was systematically collected, whereas limb events were collected on a yearly basis during follow‐up visits, precluding assignment of a precise date. Therefore, systemic and limb ischemic outcomes were analyzed separately.

The risks of CV events in each geographic region were estimated by Cox proportional hazards models adjusted for the REACH risk score predicting CV events,^12^ after exclusion of the geographic items of the score. Multivariate Cox regression models were used to assess the factors associated with CV risk in the study population. Univariate models were first built to assess the impact of each individual variable on CV outcomes. A set of variables was then selected according to their statistical significance in the univariate model (*P* ≤ 0.10), clinical significance, and nonredundancy with other variables in the model, and was introduced into the multivariate models. In addition, clinically relevant factors with plausible clinical association with ischemic events were forced into the multivariate Cox regression model.

Data were processed using the SAS software package (version 9.3; SAS Institute, Cary, NC). Elbez Yedid had access to all of the data in the study and takes responsibility for its integrity and the data analysis.

## 3 RESULTS

Overall, 65 531 patients were initially enrolled in the REACH Registry in 44 countries and 5587 centers. Of these, 8322 had a history of symptomatic PAD, of whom 6005 (76.7%, 95% confidence interval [CI]: 75.8%‐77.6%) had no history of stroke or TIA and constituted the study population for this analysis. Two‐year follow‐up was completed in 5336 (88.9%) patients, and the 4‐year follow‐up was completed in 2998 (49.9%) patients. The population included 1785 (29.7%) patients from North America, 162 (2.7%) from Latin America, 2702 (45%) from Western Europe, 441 (7.3%) from Eastern Europe, 38 (0.6%) from the Middle East, 213 (3.6%) from Asia, and 473 (7.9%) from Japan.

### 3.1 Baseline characteristics

In the overall population, the mean age was 70 ± 10 years, and 69.2% were men. At enrollment, 47.3% had diabetes mellitus, 66.2% had hypercholesterolemia, 89.1% had hypertension, 20.9% were current smokers, and 18.7% were overweight/obese. Important differences in baseline characteristics were observed according to geographic region (Table 1).

**Table 1:** Baseline patient demographics and clinical characteristics

	PAD Patients Without History of Stroke /TIA							
	North America, n = 1785	Latin America, n = 162	Western EU, n = 2702	Eastern EU, n = 441	Middle East, n = 38	Asia, n = 213	Japan, n = 473	Total, N = 6,005
Age, y, mean (SD)	70.44 (10.1)	68.73 (10.73)	68 (9.71)	62.92 (8.65)	66.14 (10.84)	66.22 (9.27)	72.03 (8.04)	70.52 (9.52)
Median	71.3	69.1	68.5	63.5	66.8	66.7	73.0	71.39
Range, q1–q3	63.3–78.7	62.4 –76.9	61.0–75.0	56.4–68.6	54.7–74.7	58.8–72.8	67.6–77.5	64.01–77.29
Men	1109 (62.1%)	100 (62.5%)	2057 (76.2%)	346 (78.5%)	30 (78.9%)	139 (65.3%)	394 (83.3%)	1261 (69.2%)
Diabetes	897 (50.5%)	96 (59.3%)	975 (36.4%)	152 (34.6%)	20 (52.6%)	148 (69.5%)	177 (37.4%)	855 (47.3%)
Hypertension	1540 (86.3%)	136 (84%)	2000 (74%)	325 (73.7%)	32 (84.2%)	166 (77.9%)	362 (76.5%)	1625 (89.1%)
Dyslipidemia	1437 (80.6%)	74 (45.7%)	1750 (64.8%)	237 (53.9%)	28 (73.7%)	133 (62.4%)	166 (35.1%)	1205 (66.2%)
Renal impairment	69 (4.6%)	4 (4%)	35 (1.9%)	4 (1%)	2 (7.1%)	18 (10.9%)	31 (7.9%)	53 (3.7%)
Angina								
Stable angina	502 (28.7%)	28 (17.7%)	639 (23.9%)	181 (41.3%)	10 (26.3%)	47 (22.6%)	78 (16.6%)	692 (38.7%)
Unstable angina	214 (12.2%)	16 (10.1%)	195 (7.3%)	68 (15.6%)	4 (10.5%)	25 (12%)	24 (5.1%)	358 (20.1%)
Polyvascular disease^a^	1072 (60.1%)	61 (37.7%)	1196 (44.3%)	260 (59%)	22 (57.9%)	97 (45.5%)	134 (28.3%)	1824 (100%)
History of MI								
≤1 year	109 (6.2%)	4 (2.6%)	110 (4.1%)	41 (9.3%)	2 (5.4%)	11 (5.3%)	8 (1.7%)	122 (6.8%)
>1 year	473 (26.9%)	26 (17%)	517 (19.3%)	104 (23.6%)	10 (27%)	24 (11.6%)	43 (9.1%)	470 (26.2%)
Atrial fibrillation/flutter	218 (12.4%)	5 (3.2%)	250 (9.5%)	52 (11.9%)	2 (5.4%)	9 (4.3%)	35 (7.4%)	290 (16.3%)
Congestive heart failure	372 (21.1%)	8 (5%)	341 (12.9%)	57 (13.1%)	7 (18.4%)	27 (12.9%)	27 (5.7%)	407 (22.9%)
Obesity								
Overweight, BMI, 25– > 30	677 (52.2%)	71 (74.7%)	1199 (67.5%)	178 (61.6%)	19 (65.5%)	61 (79.2%)	107 (93%)	717 (63.8%)
Class I, BMI, 30– > 35	396 (30.5%)	22 (23.2%)	460 (25.9%)	96 (33.2%)	7 (24.1%)	14 (18.2%)	8 (7%)	278 (24.7%)
Class II, BMI, 35– > 40	145 (11.2%)	1 (1.1%)	93 (5.2%)	13 (4.5%)	2 (6.9%)	2 (2.6%)	0 (0%)	89 (7.9%)
Class III, BMI ≥40	79 (6.1%)	1 (1.1%)	24 (1.4%)	2 (0.7%)	1 (3.4%)	0 (0%)	0 (0%)	40 (3.6%)
Smoker								
Former	962 (54.9%)	81 (52.3%)	1333 (51.1%)	168 (38.2%)	10 (28.6%)	85 (40.3%)	280 (61.4%)	859 (49%)
Current	395 (22.6%)	24 (15.5%)	741 (28.4%)	172 (39.1%)	14 (40%)	27 (12.8%)	98 (21.5%)	366 (20.9%)
Medication								
Acetylsalicylic acid	1303 (73.1%)	125 (77.2%)	1536 (57%)	328 (74.4%)	30 (78.9%)	118 (55.4%)	166 (35.1%)	1111 (61.4%)
At least 1 antiplatelet	1456 (81.6%)	138 (85.2%)	2170 (80.4%)	383 (86.8%)	35 (92.1%)	164 (77%)	384 (81.2%)	1513 (83.2%)
Angiotensin converting enzyme inhibitors	815 (46%)	58 (36.3%)	1097 (40.8%)	266 (60.3%)	17 (45.9%)	65 (30.5%)	88 (18.6%)	909 (50.4%)
Angiotensin II receptor antagonists	448 (25.4%)	30 (18.8%)	493 (18.4%)	13 (3%)	8 (21.6%)	64 (30.2%)	139 (29.4%)	360 (20.1%)
Nitrates/other anti‐angina	371 (21.4%)	22 (13.8%)	511 (19.3%)	156 (35.5%)	9 (24.3%)	52 (24.6%)	97 (20.5%)	493 (27.7%)
Statin	1371 (76.9%)	72 (44.4%)	1673 (62%)	242 (54.9%)	28 (73.7%)	134 (62.9%)	143 (30.2%)	1192 (65.5%)
β‐Blockers	943 (53.1%)	35 (21.7%)	1002 (37.3%)	203 (46%)	15 (40.5%)	80 (37.6%)	60 (12.7%)	731 (40.5%)

Abbreviations: BMI, body mass index; EU, Europe; MI, myocardial infarction; PAD, peripheral artery disease; SD, standard deviation; TIA, transient ischemic attack.

a Polyvascular disease was defined as coexistent symptomatic (clinically recognized) arterial disease in 2 or 3 territories (coronary, cerebral, and/or peripheral) within each patient.

### 3.2 Systemic ischemic events

**Figure 1:**
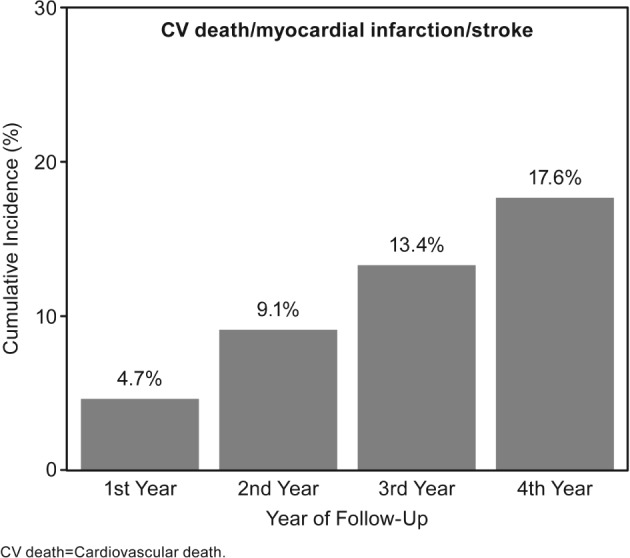
Cumulative incidence rates of primary outcome of CV death, MI, or stroke for post‐MI patients with no history of TIA/stroke. Abbreviations: CV, cardiovascular; MI, myocardial infarction; TIA, transient ischemic attack

**Figure 2:**
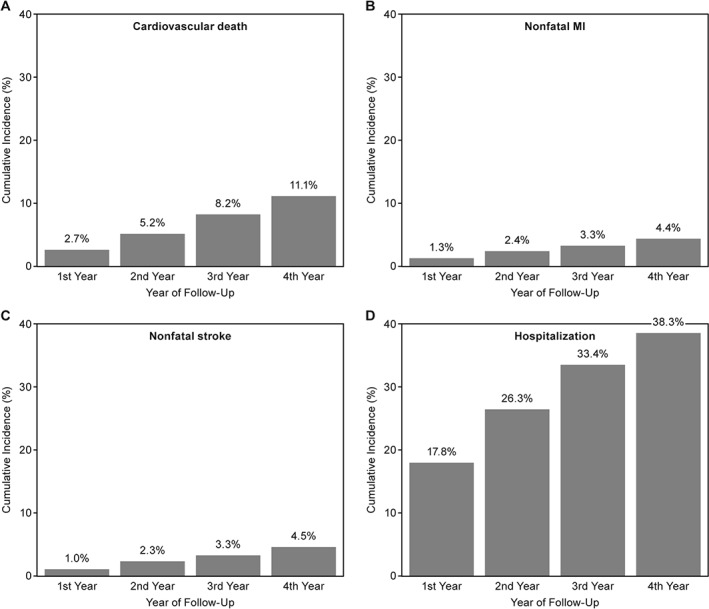
Cumulative incidence rates of cardiovascular outcomes in PAD patients with no history of stroke or TIA by year of follow‐up. Abbreviations: MI, myocardial infarction; PAD, peripheral artery disease; TIA, transient ischemic attack

The 2‐year rate of systemic ischemic events (CV death, MI, or stroke) in the overall population was 9.1%, and the 4‐year rate was 17.6% (Figure 1). The rate of systemic ischemic events increased cumulatively by approximately 4% to 5% for each year of follow‐up (Figure 1). The primary outcome was mainly driven by CV death, accounting for half of the composite outcome, with an increase of ~3% each year (2.7%, 5.2%, 8.2%, 11.1%) (Figure 2A). Nonfatal MI increased from 1.3% to 2.4% to 3.3% to 4.4% (Figure 2B) with each additional year of follow‐up, and nonfatal stroke from 1.0% to 2.3% to 3.3 to 4.5% (Figure 2C). Similarly, the rate of CV hospitalization increased cumulatively over the 4 years of follow‐up from 17.8% the first year to 26.3% to 33.4% and 38.3% the fourth year (Figure 2D).

### 3.3 According to geographic region

**Figure 3:**
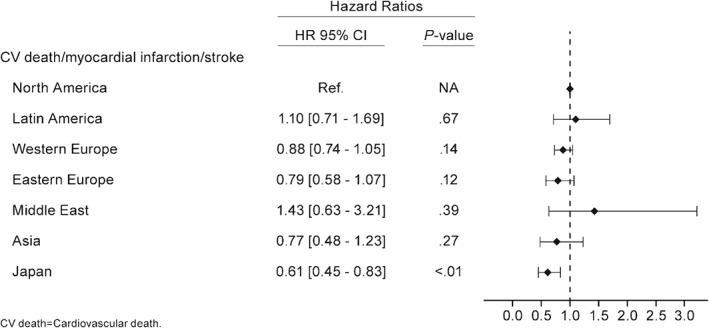
Hazard ratio for the primary outcome of cardiovascular death, MI, or stroke for post‐MI patients with no history of TIA/stroke according to geographic regions after adjustment with the REACH risk score. Abbreviations: CI, confidence interval; CV, cardiovascular; HR, hazard ratio; MI, myocardial infarction; NA, not applicable; TIA, transient ischemic attack

Compared with North America, patients who were enrolled in Eastern Europe (hazard ratio [HR] = 0.73, 95% CI: 0.55‐0.98, *P* = 0.03), Western Europe (HR = 0.83, 95% CI: 0.70‐0.98, *P* = 0.02), or Japan (HR = 0.53, 95% CI: 0.41‐0.67, *P* < 0.01) had lower crude 4‐year cumulative event rates (Figure 3). After adjusting for baseline characteristics, the only persistent difference was for Japanese patients, who experienced fewer ischemic events (HR = 0.61, 95% CI: 0.45‐0.83, *P* < 0.01) (Figure 3). Complete analysis of outcomes according to geographic region are shown in Figure 3.

### 3.4 Risk factors associated with systemic ischemic events

**Figure 4:**
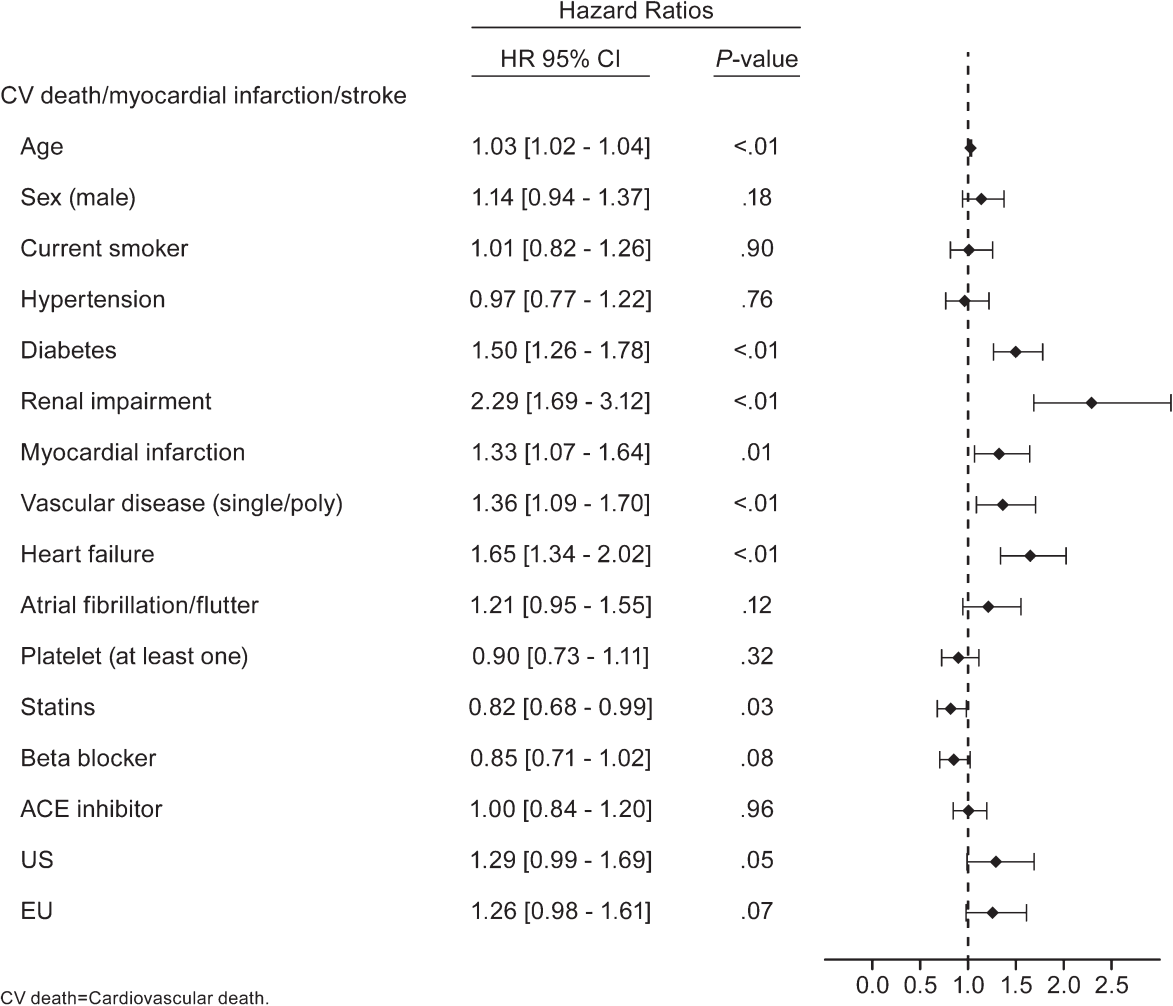
Hazard ratios of determinants for the primary outcome of CV death, nonfatal MI, and nonfatal stroke estimated by multivariate Cox models in PAD patients with no history of TIA/stroke. Abbreviations: ACE, angiotensin‐converting enzyme; CI, confidence interval; CV, cardiovascular; EU, Europe; HR, hazard ratio; MI, myocardial infarction; PAD, peripheral artery disease; TIA, transient ischemic attack; US, United States

Renal impairment (HR = 2.98, 95% CI: 1.69‐3.12, *P* < 0.01), heart failure (HR = 1.65, 95% CI: 1.34‐2.02, *P* < 0.01), history of diabetes (HR = 1.50, 95% CI: 1.26‐1.78, *P* < 0.01), history of MI (HR = 1.33, 95% CI: 1.07‐1.64, *P* = 0.01), vascular disease (single or poly; HR = 1.36, 95% CI: 1.09‐1.70, *P* < 0.01), and older age (per additional year; HR = 1.03, 95% CI: 1.02‐1.04, *P* < 0.01) were associated with increased systemic ischemic events (Figure 3). No sex or race/ethnicity associations were observed. Statin use was associated with decreased systemic ischemic risk (HR = 0.82, 95% CI: 0.69‐0.99, *P* = 0.03) (Figure 4).

### 3.5 Limb ischemic events

The 2‐year rate of the composite endpoint of limb ischemic events was 5.7%. Angioplasty and/or stenting for PAD accounted for 3.2% of these events, peripheral bypass graft accounted for 2.1%, and lower limb amputation accounted for 1.3% (patients could experience more than 1 event, explaining why the total was lower than the sum of each event). Overall, the composite outcome of systemic and limb ischemic events was 11.9% at 2 years.

## 4 DISCUSSION

This contemporary and geographically diverse study of patients with symptomatic PAD in routine clinical practice confirmed that the ischemic event rate remains high, with a cumulative 4‐year systemic event risk of 17.6%, mainly driven by CV mortality. The rate increased over follow‐up (~4%–5% per year), and there were no major differences observed across the geographic regions except for Japan, where patients experienced a lower rate of ischemic events over time. PAD patients also experienced relatively high rates of severe limb ischemic events (approximately 6% at 2 years, including a 1.3% rate of major amputations). Independent risk factors associated with the increased systemic ischemic event rate were renal impairment, heart failure, single or poly vascular disease, a history of MI, a history of diabetes, and older age. Statin use was the only factor associated with a decreased risk of recurrent ischemic events over 4 years.

The factors associated with increased ischemic events observed in this analysis were consistent with the usual CV risk factors for atherosclerotic disease.^2^, ^13^, ^14^ A previous analysis of the REACH Registry^9^ and other studies^15^, ^16^, ^17^, ^18^ suggested that patients with PAD do not achieve CV risk factor control as frequently as patients with other established atherothrombotic diseases, such as cerebrovascular disease or CV disease. In the REACH Registry, PAD patients with 3 to 5 controlled risk factors had fewer major CV events (ie, MI, stroke, CV death) compared with PAD patients with poor control (0–2 risk factors controlled, 2.66% vs. 3.52% 1‐year rates, *P* = 0.17).^19^, ^20^

In the present analysis, use of statin therapy was associated with reduced risk of ischemic events. European and US guidelines recommend low‐density lipoprotein (LDL) cholesterol reduction in patients at high CV risk.^21^, ^22^ A previous analysis of the REACH Registry showed a reduction of adverse limb outcomes in patients treated with statins for symptomatic PAD as well as overall ischemic events.^23^ The addition of more potent treatment regimens and more aggressive reduction of LDL cholesterol could provide even greater risk reduction. One study that assessed a composite outcome of coronary heart disease, cerebrovascular disease, and peripheral vascular disease events demonstrated that both statins and ezetimibe were associated with reductions in the risk of the composite outcome.^24^ In addition, the new PCSK9 inhibitors, such as alirocumab or evolocumab, showed a reduction in LDL cholesterol levels when added to maximally tolerated statin therapy. Post hoc analyses have also suggested a reduction in CV event rates in high‐risk patients with established CV disease receiving these agents,^25^, ^26^ but results from ongoing clinical trials are needed,^27^, ^28^ and these agents have yet to be assessed in the specific population of PAD patients.

Residual ischemic risk was uniformly distributed over the different geographic areas, except for Japan, where patients experienced lower event rates. The explanations for such differences have been described previously.^29^ Briefly, differences in disease management and medication use have been reported (in particular, the use of clopidogrel is substantially higher in Japan than in other regions of the world), which may have contributed to some extent in the improved outcomes in Japan, supported by evidence that clopidogrel use may be superior to aspirin in reducing major CV events in PAD patients.^30^, ^31^ In addition, gaps in country‐based payer policies and healthcare systems might explain differences in risk factor prevalence and management. Moreover, genetic susceptibilities and lifestyle differences may also play a role in risk variation.^29^ Nevertheless, residual risk remains high, and events accrued progressively over time across all geographic areas.

Antiplatelet agents are a cornerstone of the treatment of atherothrombotic disease. Patients with previous stroke or TIA were excluded from the present analysis, as they present a specific risk‐to‐benefit balance of antithrombotic use.^10^ However, antiplatelet agents were not associated with a statistically significant reduction in risk of systemic ischemic events in the present analysis. This may be related to the relatively lower efficacy of aspirin compared with clopidogrel or other antiplatelet agents in patients with PAD,^32^, ^33^ the high proportion of patients in the REACH Registry receiving aspirin as the antiplatelet agent, or that the analysis was not powered to show statistical significance.

More intensive antiplatelet treatment strategies might provide additional benefit in patients with PAD. A post hoc analysis of data from the CHARISMA (Clopidogrel for High Atherothrombotic Risk and Ischemic Stabilization, Management, and Avoidance) trial in a subgroup of patients diagnosed with PAD showed a trend for a benefit of clopidogrel plus aspirin over aspirin alone in this population, with a reduction in MI rates.^19^, ^34^ At the time the REACH Registry was established, the novel oral antithrombotic agents prasugrel, ticagrelor, vorapaxar, or rivaroxaban were not available. Therefore, the present analysis cannot provide insights on the benefit of those agents.

Results of the EUCLID (Examining Use of tiCagreLor In paD) trial, which compared monotherapy with ticagrelor or clopidogrel in PAD patients without indication for dual antiplatelet therapy, did not show a reduction in composite ischemic or limb events or in major bleeding, although a reduction in ischemic stroke was observed.^35^ Of note, patients who were homozygous for loss of function alleles to clopidogrel were excluded, though this exclusion did not seem to be the cause of the overall neutral results for ticagrelor versus clopidogrel. Therefore, all patients included in the EUCLID trial were receiving effective antiplatelet therapy.^35^

The use of a dual antiplatelet therapy rather than a single antiplatelet therapy might provide additional benefit for PAD patients. A subgroup analysis of the PLATO (Study of PLATelet inhibition and patient Outcomes) trial showed that the benefit of ticagrelor over clopidogrel in acute coronary syndrome patients was consistent in the subgroup of PAD patients compared with the global population.^36^ A similar analysis from the post‐MI PEGASUS (PEGASUS‐TIMI 54) trial also showed a consistent benefit of ticagrelor over placebo in addition to aspirin in post‐MI patients with PAD.^37^, ^38^ Similarly, a recent analysis from the DAPT (The Dual Antiplatelet Therapy Study) trial showed clear benefit of prolonged dual antiplatelet therapy in PAD patients treated with coronary stents.^39^ Recently, vorapaxar, a PAR1 platelet receptor antagonist has been evaluated in addition to aspirin in the TRA 2°P –TIMI 50 (Thrombin Receptor Antagonist in Secondary Prevention of Atherothrombotic Ischemic Events) trial in a secondary prevention setting in patients with stable atherosclerosis (defined as prior MI or stroke within the previous 2 weeks to 12 months prior to randomization) or PAD (defined as claudication and abnormal ABI or prior revascularization). In that trial, vorapaxar demonstrated a reduction in ischemic events at 3 years, including hospitalization for acute limb ischemia and peripheral revascularization.^40^ Another strategy to increase antithrombotic intensity could be the addition of an anticoagulant to aspirin. The COMPASS (Cardiovascular OutcoMes for People Using Anticoagulation StrategieS) trial (NCT01776424) assesses the effect of the addition of rivaroxaban to aspirin versus rivaroxaban alone or aspirin alone in patients with established CAD or PAD, and will provide information regarding potential benefits and risks of this approach.

There are some limitations to the present analysis. The collection of limb events and systemic events was not done in the exact same method, and there was no assignment of a precise date for limb events. Additionally, limb events were tracked on a declarative basis at the end of follow‐up, leading to some missing data at the 4‐year time point. Consequently, our analysis was underpowered to determine risk factors for these occurrences. These analyses are drawn from an observational registry; the results presented here are therefore descriptive, and analyses on the determinants of residual risk as well as analyses on geographic differences must be interpreted cautiously. Clinical events were not adjudicated in the REACH Registry, but measures were taken to select high‐quality physicians, and diagnoses were provided by hospitals and doctors based on their expertise. Patient adherence to medication was not captured in the registry, and adherence could impact patient outcomes. Finally, although the data were taken from a large cohort, the analysis may have been underpowered for some comparisons.

## 5 CONCLUSION

This analysis of the REACH Registry showed an important ischemic risk in patients with PAD, continuously increasing over the 4 years of follow‐up and mainly driven by CV mortality. Limb ischemic events represent a substantial additional burden to that related to systemic ischemic events in this population. Secondary prevention strategies, including enhanced antithrombotic treatment and more intense lipid lowering, may be useful to improve prevention of ischemic and limb vascular events in this high‐risk population.

### Author Contributions

All authors meet the criteria for authorship stated in the Uniform Requirements for Manuscripts Submitted to Biomedical Journals. Specifically, all authors made substantial contributions to the conception and design of the study and manuscript or interpretation of the data, contributed to the drafting of the article or revising it critically for important intellectual content, and all gave final approval of the version to be published. In addition, all authors agree to be accountable for all aspects of the work in ensuring that questions related to the accuracy or integrity of any part of the work are appropriately investigated and resolved.

### Conflicts of Interest

Dr. Deepak L. Bhatt discloses the following relationships: Advisory Board: Cardax, Elsevier Practice Update Cardiology, Medscape Cardiology, Regado Biosciences; Board of Directors: Boston VA Research Institute, Society of Cardiovascular Patient Care; Chair: American Heart Association Quality Oversight Committee; Data Monitoring Committees: Duke Clinical Research Institute, Harvard Clinical Research Institute, Mayo Clinic, Population Health Research Institute; Honoraria: American College of Cardiology (Senior Associate Editor, Clinical Trials and News, ACC.org), Belvoir Publications (Editor in Chief, *Harvard Heart Letter*), Duke Clinical Research Institute (clinical trial steering committees), Harvard Clinical Research Institute (clinical trial steering committee), HMP Communications (Editor in Chief, *Journal of Invasive Cardiology*), *Journal of the American College of Cardiology* (Guest Editor; Associate Editor), Population Health Research Institute (clinical trial steering committee), Slack Publications (Chief Medical Editor, *Cardiology Today's Intervention*), Society of Cardiovascular Patient Care (Secretary/Treasurer), WebMD (CME steering committees); Other: *Clinical Cardiology* (Deputy Editor), NCDR‐ACTION Registry Steering Committee (Chair), VA CART Research and Publications Committee (Chair); Research Funding: Amarin, Amgen, AstraZeneca, Bristol‐Myers Squibb, Chiesi, Eisai, Ethicon, Forest Laboratories, Ischemix, Lilly, Medtronic, Pfizer, Roche, Sanofi Aventis, The Medicines Company; Royalties: Elsevier (Editor, *Cardiovascular Intervention: A Companion to Braunwald's Heart Disease*); Site coinvestigator: Biotronik, Boston Scientific, St. Jude Medical; Trustee: American College of Cardiology; Unfunded Research: FlowCo, PLx Pharma, Takeda. Dr. Philippe Gabriel Steg discloses the following relationships: research grant from Merck, Sanofi, and Servier; speaking or consulting fees from Amarin, AstraZeneca, Bayer, Boehringer‐Ingelheim, Bristol‐Myers‐Squibb, CSL‐Behring, Daiichi‐Sankyo, GlaxoSmithKline, Janssen, Lilly, Merck, Novartis, Pfizer, Regeneron, Roche, Sanofi, Servier, The Medicines Company. Dr. David Wu, Dr. Mary E. Hanson, Dr. Hakima Hannachi, and Dr. Puneet K. Singhal are employees of Merck & Co., Inc., and own stock and/or stock options in the company. Dr. Gregory Ducrocq discloses the following relationships: speaker's and/or consulting fees from Astra Zeneca, Biotronik, BMIS, Daiichi Sankyo and Lilly; Advisory Board: Lilly; CEC: Sanofi and Phillips; DSMB: Abbot and MicroPort; and travel fees from Astra Zeneca.

A full list of the REACH Registry investigators can be found in Bhatt DL, Steg PG, Ohman EM, et al. International prevalence, recognition, and treatment of cardiovascular risk factors in outpatients with atherothrombosis. *JAMA*. 2006;295:180–189.

## References

[1] Fowkes FG , Rudan D , Rudan I , et al. Comparison of global estimates of prevalence and risk factors for peripheral artery disease in 2000 and 2010: a systematic review and analysis. Lancet. 2013;382:1329–1340. 23915883 10.1016/S0140-6736(13)61249-0

[2] Tendera M , Aboyans V , Bartelink ML , et al. ESC Guidelines on the diagnosis and treatment of peripheral artery diseases: document covering atherosclerotic disease of extracranial carotid and vertebral, mesenteric, renal, upper and lower extremity arteries: the Task Force on the Diagnosis and Treatment of Peripheral Artery Diseases of the European Society of Cardiology (ESC). Eur Heart J. 2011;32:2851–2906. 21873417 10.1093/eurheartj/ehr211

[3] Violi F , Basili S , Berger JS , et al. Antiplatelet therapy in peripheral artery disease. Handb Exp Pharmacol. 2012;(210):547–563. 10.1007/978-3-642-29423-5_22 22918746

[4] Criqui MH , Langer RD , Fronek A , et al. Mortality over a period of 10 years in patients with peripheral arterial disease. N Engl J Med. 1992;326:381–386. 1729621 10.1056/NEJM199202063260605

[5] Rothwell PM , Coull AJ , Silver LE , et al. Population‐based study of event‐rate, incidence, case fatality, and mortality for all acute vascular events in all arterial territories (Oxford Vascular Study). Lancet. 2005;366:1773–1783. 16298214 10.1016/S0140-6736(05)67702-1

[6] Steg PG , Bhatt DL , Wilson PW , et al. One‐year cardiovascular event rates in outpatients with atherothrombosis. JAMA. 2007;297:1197–1206. 17374814 10.1001/jama.297.11.1197

[7] Bhatt DL , Steg PG , Ohman EM , et al. International prevalence, recognition, and treatment of cardiovascular risk factors in outpatients with atherothrombosis. JAMA. 2006;295:180–189. 16403930 10.1001/jama.295.2.180

[8] Bhatt DL , Eagle KA , Ohman EM , et al. Comparative determinants of 4‐year cardiovascular event rates in stable outpatients at risk of or with atherothrombosis. JAMA. 2010;304:1350–1357. 20805624 10.1001/jama.2010.1322

[9] Cacoub PP , Abola MT , Baumgartner I , et al. Cardiovascular risk factor control and outcomes in peripheral artery disease patients in the Reduction of Atherothrombosis for Continued Health (REACH) Registry. Atherosclerosis. 2009;204:e86–e92. 19054514 10.1016/j.atherosclerosis.2008.10.023

[10] Ducrocq G , Amarenco P , Labreuche J , et al. A history of stroke/transient ischemic attack indicates high risks of cardiovascular event and hemorrhagic stroke in patients with coronary artery disease. Circulation. 2013;127:730–738. 23277306 10.1161/CIRCULATIONAHA.112.141572

[11] Ohman EM , Bhatt DL , Steg PG , et al. The REduction of Atherothrombosis for Continued Health (REACH) Registry: an international, prospective, observational investigation in subjects at risk for atherothrombotic events‐study design. Am Heart J . 2006;151:786 786.e1–e10. 16569533 10.1016/j.ahj.2005.11.004

[12] Wilson PW , D'Agostino R Sr , Bhatt DL , et al. An international model to predict recurrent cardiovascular disease. Am J Med. 2012;125:695–703. 22727237 10.1016/j.amjmed.2012.01.014

[13] Bartholomew JR , Olin JW . Pathophysiology of peripheral arterial disease and risk factors for its development. Cleve Clin J Med . 2006;73(suppl 4):S8–S14. 10.3949/ccjm.73.suppl_4.s8 17385386

[14] Shanmugasundaram M , Ram VK , Luft UC , et al. Peripheral arterial disease—what do we need to know? Clin Cardiol. 2011;34:478–482. 21717473 10.1002/clc.20925 PMC6652699

[15] Blacher J , Cacoub P , Luizy F , et al. Peripheral arterial disease versus other localizations of vascular disease: the ATTEST study. J Vasc Surg. 2006;44:314–318. 16890860 10.1016/j.jvs.2006.04.002

[16] Blanes JI , Cairols MA , Marrugat J . Prevalence of peripheral artery disease and its associated risk factors in Spain: the ESTIME Study. Int Angiol. 2009;28:20–25. 19190551

[17] Hirsch AT , Criqui MH , Treat‐Jacobson D , et al. Peripheral arterial disease detection, awareness, and treatment in primary care. JAMA. 2001;286:1317–1324. 11560536 10.1001/jama.286.11.1317

[18] Makdisse M , Ramos LR , Moreira F , et al. A risk score for predicting peripheral arterial disease in individuals 75 years or older. Arq Bras Cardiol. 2007;88:630–636. 17664989 10.1590/s0066-782x2007000600002

[19] Cacoub PP , Bhatt DL , Steg PG , et al. Patients with peripheral arterial disease in the CHARISMA trial. Eur Heart J. 2009;30:192–201. 19136484 10.1093/eurheartj/ehn534

[20] Abola MT , Bhatt DL , Duval S , et al. Fate of individuals with ischemic amputations in the REACH Registry: three‐year cardiovascular and limb‐related outcomes. Atherosclerosis. 2012;221:527–535. 22321872 10.1016/j.atherosclerosis.2012.01.002

[21] Reiner Z , Catapano AL , De Backer G , et al. ESC/EAS guidelines for the management of dyslipidaemias: the Task Force for the Management of Dyslipidaemias of the European Society of Cardiology (ESC) and the European Atherosclerosis Society (EAS). Eur Heart J. 2011;32:1769–1818. 21712404 10.1093/eurheartj/ehr158

[22] Stone NJ , Robinson J , Lichtenstein AH , et al. 2013 ACC/AHA guideline on the treatment of blood cholesterol to reduce atherosclerotic cardiovascular risk in adults: a report of the American College of Cardiology/American Heart Association Task Force on practice guidelines. J Am Coll Cardiol . 2014;63(25 pt B):2889–2934. 24239923 10.1016/j.jacc.2013.11.002

[23] Kumbhani DJ , Steg PG , Cannon CP , et al. Statin therapy and long‐term adverse limb outcomes in patients with peripheral artery disease: insights from the REACH registry. Eur Heart J. 2014;35:2864–2872. 24585266 10.1093/eurheartj/ehu080 PMC4216432

[24] Hayek S , Canepa EF , Sattar A , et al. Effect of ezetimibe on major atherosclerotic disease events and all‐cause mortality. Am J Cardiol. 2013;111:532–539. 23219178 10.1016/j.amjcard.2012.11.002 PMC3563770

[25] Robinson JG , Farnier M , Krempf M , et al. Efficacy and safety of alirocumab in reducing lipids and cardiovascular events. N Engl J Med. 2015;372:1489–1499. 25773378 10.1056/NEJMoa1501031

[26] Sabatine MS , Giugliano RP , Wiviott SD , et al. Efficacy and safety of evolocumab in reducing lipids and cardiovascular events. N Engl J Med. 2015;372:1500–1509. 25773607 10.1056/NEJMoa1500858

[27] Sabatine MS , Giugliano RP , Keech A , et al. Rationale and design of the Further cardiovascular OUtcomes Research with PCSK9 Inhibition in subjects with Elevated Risk trial. Am Heart J. 2016;173:94–101. 26920601 10.1016/j.ahj.2015.11.015

[28] Schwartz GG , Bessac L , Berdan LG , et al. Effect of alirocumab, a monoclonal antibody to PCSK9, on long‐term cardiovascular outcomes following acute coronary syndromes: rationale and design of the ODYSSEY outcomes trial. Am Heart J. 2014;168:682–689. 25440796 10.1016/j.ahj.2014.07.028

[29] Ducrocq G , Bhatt DL , Labreuche J , et al. Geographic differences in outcomes in outpatients with established atherothrombotic disease: results from the REACH Registry. Eur J Prev Cardiol. 2014;21:1509–1516. 23965467 10.1177/2047487313501278

[30] Katsanos K , Spiliopoulos S , Saha P , et al. Comparative efficacy and safety of different antiplatelet agents for prevention of major cardiovascular events and leg amputations in patients with peripheral arterial disease: a systematic review and network meta‐analysis. PLoS One. 2015;10:e0135692. 10.1371/journal.pone.0135692 PMC4537264 26274912

[31] Bhatt DL , Fox KA , Hacke W , et al. Clopidogrel and aspirin versus aspirin alone for the prevention of atherothrombotic events. N Engl J Med. 2006;354:1706–1717. 16531616 10.1056/NEJMoa060989

[32] Baigent C , Blackwell L , Collins R , et al. Aspirin in the primary and secondary prevention of vascular disease: collaborative meta‐analysis of individual participant data from randomised trials. Lancet. 2009;373:1849–1860. 19482214 10.1016/S0140-6736(09)60503-1 PMC2715005

[33] CAPRIE Steering Committee . A randomised, blinded, trial of clopidogrel versus aspirin in patients at risk of ischaemic events (CAPRIE). Lancet . 1996;348:1329–1339. 8918275 10.1016/s0140-6736(96)09457-3

[34] Bhatt DL , Flather MD , Hacke W , et al. Patients with prior myocardial infarction, stroke, or symptomatic peripheral arterial disease in the CHARISMA trial. J Am Coll Cardiol. 2007;49:1982–1988. 17498584 10.1016/j.jacc.2007.03.025

[35] Hiatt WR , Fowkes FG , Heizer G , et al. Ticagrelor versus clopidogrel in symptomatic peripheral artery disease. N Engl J Med. 2017;376:32–40. 27959717 10.1056/NEJMoa1611688

[36] Patel MR , Becker RC , Wojdyla DM , et al. Cardiovascular events in acute coronary syndrome patients with peripheral arterial disease treated with ticagrelor compared with clopidogrel: data from the PLATO Trial. Eur J Prev Cardiol. 2015;22:734–742. 24830710 10.1177/2047487314533215

[37] Bonaca MP , Bhatt DL , Storey RF , et al. Ticagrelor for prevention of ischemic events after myocardial infarction in patients with peripheral artery disease. J Am Coll Cardiol. 2016;67:2719–2728. 27046162 10.1016/j.jacc.2016.03.524

[38] Bonaca MP , Bhatt DL , Cohen M , et al. Long‐term use of ticagrelor in patients with prior myocardial infarction. N Engl J Med. 2015;372:1791–1800. 25773268 10.1056/NEJMoa1500857

[39] Mauri L , Kereiakes DJ , Yeh RW , et al. Twelve or 30 months of dual antiplatelet therapy after drug‐eluting stents. N Engl J Med. 2014;371:2155–2166. 25399658 10.1056/NEJMoa1409312 PMC4481318

[40] Morrow DA , Braunwald E , Bonaca MP , et al. Vorapaxar in the secondary prevention of atherothrombotic events. N Engl J Med. 2012;366:1404–1413. 22443427 10.1056/NEJMoa1200933

